# Surgical Management of Cervical Myelopathy Due to Congenital Synostosis at the Craniovertebral Junction: Vertebral Artery Anomalies and Full-Endoscopic Retropharyngeal Odontoidectomy in a Case Series of Five Patients

**DOI:** 10.7759/cureus.98143

**Published:** 2025-11-30

**Authors:** Hiroshi Kageyama, Yukoh Ohara, Hirohiko Arimoto, Toru Maeda, Koichi Sairyo

**Affiliations:** 1 Orthopedics, Institute of Biomedical Sciences, Tokushima University Graduate School, Tokushima, JPN; 2 Neurological Surgery, Shin-Kuki General Hospital, Kuki, JPN; 3 Orthopedics, Anan Medical Center, Anan, JPN; 4 Neurosurgery, Juntendo University, Tokyo, JPN

**Keywords:** craniovertebral junction, decompression, endoscope, myelopathy, synostosis, vertebral artery

## Abstract

Congenital synostosis at the craniovertebral junction (CVJ), including C1 assimilation and C2-C3 fusion, can lead to cervical myelopathy due to adjacent segment stenosis or instability. These anomalies are rare and frequently associated with vertebral artery (VA) variations, which complicate surgical planning and instrumentation. This case report describes five patients who developed cervical myelopathy associated with congenital CVJ synostosis and were surgically treated at our institution between 2019 and 2023. Preoperative imaging, including computed tomography (CT), magnetic resonance imaging (MRI), and CT angiography, was used to evaluate bony anomalies and VA morphology. Posterior decompression and fusion were performed under navigation guidance, with screw selection tailored to the individual anatomy. Among the five patients (mean age, 74.8 years), three had C1 assimilation, four had C2-C3 fusion, and two had both. VA anomalies, such as high-riding VA and unilateral hypoplasia, were present in all patients with C2-C3 fusion, requiring modified screw trajectories to prevent vascular injury. Three patients underwent full-endoscopic retropharyngeal odontoidectomy for persistent ventral compression after posterior fixation. All patients showed postoperative neurological improvement, and no VA injury or screw malposition occurred. Imaging confirmed sufficient decompression and appropriate fixation. Congenital CVJ synostosis with associated VA anomalies presents unique surgical challenges. Preoperative vascular imaging and intraoperative navigation are essential for safe fixation, and full-endoscopic retropharyngeal odontoidectomy offers a minimally invasive and effective option for anterior decompression in selected cases.

## Introduction

Congenital anomalies at the craniovertebral junction (CVJ), which include C1 assimilation and fused vertebrae at C2/3, can lead to progressive cervical myelopathy due to mechanical compression at adjacent mobile segments. These anomalies are underrecognized and may present with diverse clinical and radiological features, complicating surgical decision-making.

C1 assimilation is an osteogenic anomaly in which the atlas (the C1 vertebra) is partially or completely fused to the occipital bone of the skull. This condition was first described by Rokitansky in 1844 and first demonstrated radiographically by Schullerin in 1911. The incidence ranges from 0.14% to 0.75% in the general population, with a reported prevalence of 0.08% to 2.76%. The fusion may be partial or complete, affecting the anterior arch, posterior arch, or both to varying degrees [1].

A clinically important morphological variant in the cervical spine is the complete or partial fusion (synostosis) of two or more cervical vertebrae, which function as a single unit. Congenital cervical synostosis is believed to result from a segmentation failure of the cervical somites during the third to eighth weeks of gestation. In this anomaly, the notochord fails to form the nucleus pulposus, leading either to a rudimentary fibrous intervertebral junction or to a complete absence of any disk-like structure between the affected vertebrae [2]. The fusion occurs most frequently between the axis and third cervical vertebrae (the C2-C3 osseous complex), with a reported prevalence ranging from 0.10% to 1.33%, followed by fusion of the fifth and sixth cervical vertebrae (C5-C6) [2].

One of the major surgical challenges in such cases is the existence of vertebral artery (VA) anomalies, particularly high-riding VAs (HRVAs) and VA hypoplasia, which may limit the feasibility and safety of conventional posterior screw placement techniques. During the fifth to eighth weeks of embryogenesis, the VAs develop as bilateral longitudinal anastomoses linking the first seven cervical intersegmental arteries [3]. Variations in this process can produce anomalies such as HRVAs and unilateral VA hypoplasia. HRVA is an anatomical variant in which the VA courses abnormally high/medial at C2, effectively reducing the bone corridor and complicating C2 instrumentation [4]. VA hypoplasia denotes underdevelopment of one VA (commonly defined as <3 mm diameter), a frequent variant with a prevalence of 1.9-26% that usually affects one side (predominantly the right) [5]. Therefore, preoperative recognition and adaptation of the instrumentation strategy are critical to avoid neurovascular complications.

Furthermore, odontoid resection is sometimes necessary for ventral spinal cord decompression. While transoral or extended approaches have traditionally been used, the endoscopic retropharyngeal approach has recently emerged as a minimally invasive alternative, offering direct access with favorable clinical outcomes.

In this case series, we describe five patients with cervical myelopathy caused by adjacent segment stenosis associated with C1 assimilation and C2/3 vertebral fusion. The two key findings in this series were (1) a high incidence of HRVAs that necessitated individualized screw placement strategies during posterior fixation and (2) decompression via full-endoscopic retropharyngeal odontoidectomy in three patients was successful, leading to favorable neurological outcomes.

## Case presentation

Patient selection

Patients who underwent spinal surgery at our institution between January 2019 and December 2023 were retrospectively reviewed. Those who presented with cervical myelopathy associated with congenital vertebral synostosis at the CVJ and upper cervical spine, specifically C1 assimilation and/or C2-C3 vertebral fusion, were included in our case series. The diagnosis of congenital vertebral fusion was made based on radiological findings without evidence of degenerative fusion. Among the 666 patients who underwent spinal surgery during the study period, five Japanese patients (4 females, 1 male) were identified as having cervical myelopathy associated with congenital vertebral synostosis (Table 1). These patients had a mean age of 74.8 years (range, 65-85). This study received approval from the Ethics Committee of Shin-Kuki General Hospital (approval No. 0116), and informed consent was obtained from all patients.

**Table 1:** Summary of clinical characteristics and surgical strategy. HRVA, high-riding vertebral artery; OC, occipitocervical.

Case	Age (years)	Sex	C1 assimilation	C2/3 fusion	VA anomaly	Fixation strategy	Odontoidectomy
1	70	F	Yes	Yes	Left HRVA, right hypoplasia	OC fusion	Yes
2	77	F	Yes	Yes	Right HRVA, left hypoplasia	OC fusion	Yes
3	85	F	No	Yes	Bilateral HRVA	C3(2)-C4 fusion	No
4	65	F	No	Yes	Left HRVA, right hypoplasia	C1–C2 fusion	Yes
5	78	M	Yes	No	None	OC fusion	No

Preoperative assessment

All patients underwent comprehensive preoperative evaluation, including clinical neurological examination and imaging studies. Radiological evaluation included plain radiographs, computed tomography (CT), and magnetic resonance images. CT angiography was routinely performed to assess the anatomy of the VA before surgical planning. A HRVA was defined as a C2 pedicle height of <5 mm or an isthmus height of <2 mm, measured 3 mm lateral to the border of the spinal canal, based on axial CT measurements. Atlantoaxial subluxation, basilar invagination, and other associated anomalies were also evaluated.

Surgical intervention

Surgical intervention was indicated for patients presenting with progressive cervical myelopathy and radiological evidence of spinal cord compression or instability. The surgical strategy was determined based on individual patient anatomy and compressive pathology: Posterior fixation and decompression were performed in all cases using occipitocervical fusion, C1-C2 fusion, or subaxial fusion depending on the instability pattern. Intraoperative three-dimensional (3D) navigation was used in all cases to assist safe screw placement. The screw type (transarticular, laminar, lateral mass, or pedicle) was selected according to the anatomy of the VA and bony morphology.

**Figure 1:**
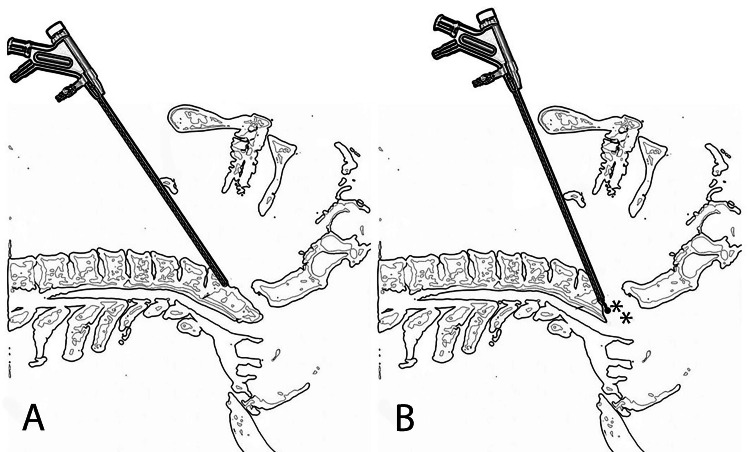
Schematic illustration of full-endoscopic uniportal retropharyngeal odontoidectomy (lateral view). (A) Before odontoid resection. (B) After odontoid resection. The resection area is indicated by asterisks.

Full-endoscopic uniportal retropharyngeal odontoidectomy was performed in selected patients requiring ventral decompression of the cervicomedullary junction under general anesthesia using a system for full-endoscopic spinal surgery with continuous irrigation (Figure 1). The patient was placed in the supine position. The procedure is performed through a small anterior neck incision, accessing the odontoid process (dens) of the C2 vertebrae via the retropharyngeal space, thus avoiding the oral or nasal cavity. An 8-mm working sleeve and endoscope are inserted to visualize and gradually remove the odontoid process with specialized instruments.

Patients were followed for at least six months postoperatively. Postoperative CT was used to evaluate screw position and bone fusion, and magnetic resonance imaging was used to assess decompression of the spinal cord.

Results

**Figure 2:**
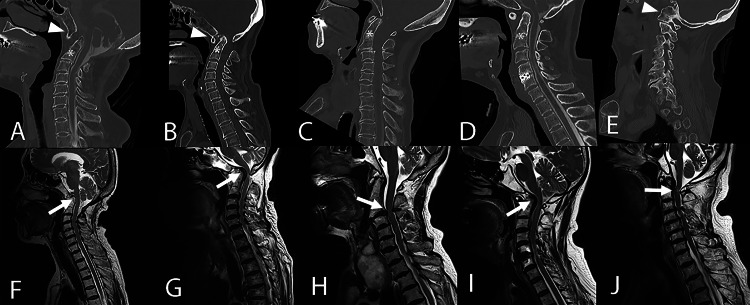
Preoperative CT or CT myelography (upper row) and magnetic resonance imaging (lower row) scans. (A, F) Case 1, (B, G) Case 2, (C, H) Case 3, (D, I) Case 4, and (E, J) Case 5. White arrow heads: C1 assimilation. Asterisks: C2-C3 vertebral fusion.  White arrows: spinal cord compression at adjacent mobile segments. CT: computed tomography.

Three cases had C1 assimilation, four had C2-C3 vertebral fusion, and two had both anomalies. All five patients presented with progressive myelopathic symptoms and radiological evidence of spinal cord compression at adjacent mobile segments (Figure 2).

Atlantoaxial subluxation or basilar invagination was observed in three of the four patients with C2-C3 fusion. Case 1 had a history of foramen magnum decompression performed approximately 50 years earlier, resulting in significant suboccipital bone loss. Case 4 had posterior arch hypoplasia resembling spina bifida.

**Figure 3:**
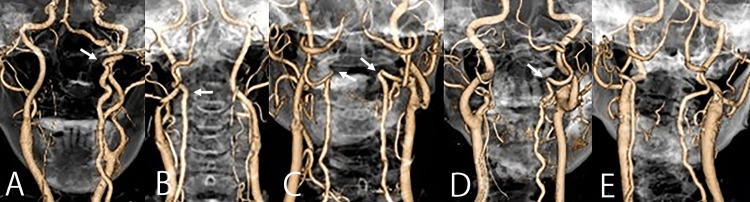
Preoperative three-dimensional CT angiography showing anterior and posterior views with bone-transparent cervical spine reconstruction. (A) Case 1, (B) Case 2, (C) Case 3, (D) Case 4, and (E) Case 5. White arrows: HRVA. HRVA: high-riding vertebral artery; CT: computed tomography.

**Figure 4:**
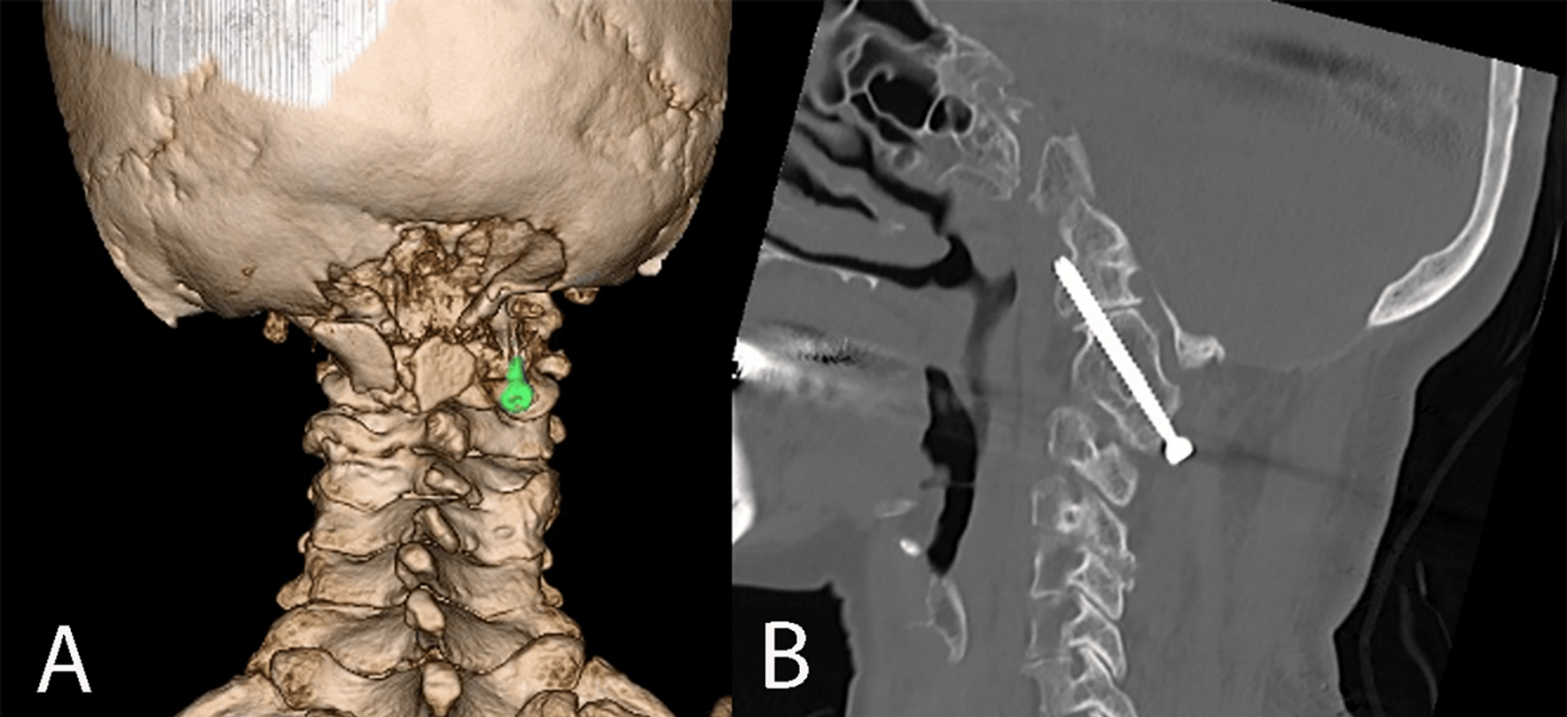
Case 1. Occipitocervical fusion with a hemilateral TAS and iliac bone grafting. (A) Three-dimensional CT; (B) CT sagittal section. TAS: transarticular screw; CT: computed tomography.

All four patients with C2-C3 fusion were found to have VA anomalies on preoperative 3D-CT angiography. Three cases had unilateral VA hypoplasia, and in each, the dominant contralateral VA followed a high-riding course. One patient had bilateral HRVAs (Table 1, Figure 3). These anatomical variations necessitated individualized screw strategies to avoid vascular injury, including the use of transarticular, laminar, pedicle, and lateral mass screws. Navigation assistance was used in all cases to ensure safety. Occipitocervical fusion was performed in three cases, C1-C2 fusion in one, and C3-C4 fusion in one. Bone grafts included autologous iliac crest and allografts (Table 2). In the patient who underwent suboccipital decompression, C1 assimilation was present. For occipitocervical fusion, a transarticular screw (TAS) was inserted between the occipital bone, C1, and C2. However, the procedure was performed unilaterally because the VA had an anomalous course. On the contralateral side, sufficient autologous bone grafting was applied, including over the facet joint (Figure 4).

**Table 2:** Summary of screw selection and type of bone graft. TAS, transarticular screw; PS, pedicle screw; LMS, lateral mass screw.

Case	Screw and bone graft
1	Left O/C2 iliac bone grafting, right occipitocervical (O/C1)/C2 TAS
2	Left C2 PS, C3 PS, C4 PS, right C2 laminar screw + C4 LMS, O/C2 iliac bone grafting
3	Bilateral C3 LMS, C4 LMS
4	Left C1 LMS, C2 laminar screw, right C1/C2 TAS. Bone grafting: McGraw
5	Bilateral C2 PS, C3 PS, O/C2 iliac bone grafting

Only the patient (Case 1) who underwent unilateral TAS fixation and bone grafting for an occipital bone defect required immobilization in a halo vest for three months. All other patients were treated with a rigid cervical collar for one month.

**Figure 5:**
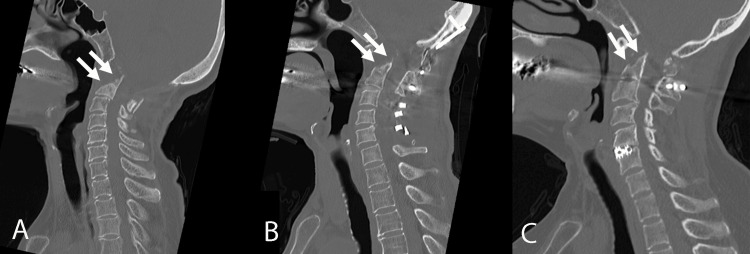
Full-endoscopic retropharyngeal uniportal anterior odontoidectomy in three cases with atlantoaxial subluxation or basilar invagination. (A) Case 1, (B) Case 2, and (C) Case 4. White arrows: Site of odontoid resection.

Three patients with atlantoaxial subluxation causing medullary and spinal cord compression underwent full-endoscopic retropharyngeal odontoidectomy one month after the initial fusion surgery because of persistent ventral compression (Figure 5). Oral intake was resumed the day after surgery, and ambulation was initiated with a cervical collar in place.

All patients improved during postoperative follow-up. No VA injuries or screw malpositioning was observed. Radiological evaluation confirmed adequate decompression and fixation.

## Discussion

Congenital vertebral synostosis in the CVJ and upper cervical spine, such as C1 assimilation and C2/3 fusion, can lead to mechanical instability and adjacent segment disease, resulting in cervical myelopathy. The clinical presentation can be delayed until later in life, as the adjacent mobile segments gradually undergo degenerative changes and compensatory hypermobility. This is consistent with a previous report demonstrating that congenital cervical fusion predisposes patients to adjacent segment degeneration and spinal cord compression at mid-cervical levels [6].

Our five patients showed neurological deterioration as a consequence of adjacent segment stenosis or instability. Notably, C1 assimilation and C2/3 fusion often coexisted with atlantoaxial subluxation or basilar invagination, reinforcing the notion that these congenital anomalies predispose to complex craniovertebral instability. This observation aligns with the reports of Tian et al. and Yamazaki et al., who found significant associations of congenital skeletal anomalies at the CVJ with mechanical strain and occipitocervical instability [7,8].

A HRVA is an anatomical variant in which the VA takes an unusual course at the level of the C2 vertebra. Specifically, the artery runs too medially, too posteriorly, and/or too superiorly within the bone, particularly at the isthmus of C2 [4]. This abnormal path results in narrowing of the safe bony corridor for placement of C2 pedicle screws during spinal surgery. On CT scans, a HRVA is typically defined by a C2 isthmus height of ≤5 mm and/or a C2 internal height of ≤2 mm measured 3 mm lateral to the border of the spinal canal [4]. Shimizu et al. defined a HRVA as having a maximum C2 pedicle diameter of <3.5 mm on axial CT [9]. A particularly important observation in our series was the high prevalence of VA anomalies. All four patients with C2/3 fusion had anomalous VA courses, including HRVAs and unilateral hypoplasia (Figure 3). These findings are consistent with findings in both Asian and European populations, including in the studies reported by Yamazaki et al., and Vaněk et al., who documented frequent HRVAs with congenital CVJ anomalies [8,10]. Furthermore, advanced age and female sex were noted to be significant risk factors for HRVA, a finding that correlates with the demographic distribution in our cohort [10].

Considering these vascular anomalies, posterior instrumentation required individualized screw selection strategies. Navigation-assisted transarticular, laminar, or lateral mass screw placement was critical for avoiding iatrogenic VA injury. The value of intraoperative navigation in such anatomically complex scenarios has been underscored in the literature, including in reports by Tian et al. and Kosnik-Infinger et al., who showed that neuronavigation significantly improves the accuracy and safety of instrumentation in the presence of VA anomalies and incomplete occipital bone [7,11].

Another key contribution of this study is the application of a full-endoscopic uniportal retropharyngeal approach for odontoidectomy in selected cases. This technique has been reported to be a minimally invasive and effective alternative to traditional transoral approaches. Ruetten et al. and Ohara et al. have demonstrated that this approach provides direct access to the odontoid process with reduced risks of infection, dysphagia, and soft tissue morbidity [12-14]. In our series, three patients underwent endoscopic odontoidectomy with favorable clinical outcomes and no perioperative complications, supporting the broader applicability of this technique.

Given the anatomical variations commonly seen in congenital CVJ anomalies, the surgeon must maintain a flexible armamentarium of fixation options. In our series, the ability to pivot between laminar, transarticular, and lateral mass screws based on preoperative imaging and intraoperative findings was paramount. This emphasizes prioritizing VA preservation over strict adherence to conventional fixation trajectories. The use of intraoperative 3D navigation facilitated this individualized planning and implementation.

While traditionally reserved for non-reducible basilar invagination, our experience suggests that full-endoscopic retropharyngeal odontoidectomy may be a viable option. The low morbidity and precise decompression afforded by this technique may justify its use earlier in the surgical algorithm, especially in elderly patients and those with multiple comorbidities. Prospective studies are needed to better define its indications and long-term results.

Given that congenital anomalies of the CVJ are rare but clinically significant, multicenter collaboration is essential to refine surgical strategies and outcome prediction. In particular, the relationship between specific fusion patterns (e.g., C1 assimilation vs. C2/3 fusion) and the distribution of HRVA should be explored further using high-resolution vascular imaging. Establishing a centralized registry of such patients may facilitate larger-scale analyses and the development of consensus-based management algorithms.

This case series is limited by its retrospective nature and small sample size. However, given the rarity of symptomatic CVJ synostosis and the technical challenges in surgical management, we believe that our findings provide important insights for operative planning, particularly regarding vascular risk and the anterior decompression strategy. The integration of high-resolution vascular imaging and minimally invasive techniques appears to be critical in the safe and effective treatment of these complex cases.

## Conclusions

Congenital synostosis at the CVJ may lead to cervical myelopathy secondary to adjacent segment stenosis or instability. HRVAs are frequently associated with these anomalies and pose significant challenges for posterior instrumentation. Thorough preoperative vascular evaluation using CT or MR angiography, combined with intraoperative navigation, is essential to ensure safe and effective fixation. Furthermore, full-endoscopic retropharyngeal odontoidectomy represents a minimally invasive and effective option for anterior decompression in selected cases, contributing to favorable neurological outcomes.

## References

[1] Hari Hara Hanusun N, Singh A, Poddar P (2024). Atlanto-occipital assimilation: embryological basis and its clinical significance. Anat Cell Biol.

[2] Demeneopoulou E, Papa D, Giotas I (2023). Fusion of the 2nd with the 3rd cervical vertebrae (C2-C3): a case series with possible clinical significance. Case Rep Orthop.

[3] Yuan SM (2016). Aberrant origin of vertebral artery and its clinical implications. Braz J Cardiovasc Surg.

[4] Klepinowski T, Żyłka N, Pala B, Poncyljusz W, Sagan L (2021). Prevalence of high-riding vertebral arteries and narrow C2 pedicles among Central-European population: a computed tomography-based study. Neurosurg Rev.

[5] Thierfelder KM, Baumann AB, Sommer WH (2014). Vertebral artery hypoplasia: frequency and effect on cerebellar blood flow characteristics. Stroke.

[6] Nouri A, Martin AR, Lange SF, Kotter MR, Mikulis DJ, Fehlings MG (2017). Congenital cervical fusion as a risk factor for development of degenerative cervical myelopathy. World Neurosurg.

[7] Tian W, Weng C, Li Q (2013). Occipital-C2 transarticular fixation for occipitocervical instability associated with occipitalization of the atlas in patients with Klippel-Feil syndrome, using intraoperative 3-dimensional navigation system. Spine (Phila Pa 1976).

[8] Yamazaki M, Okawa A, Furuya T (2012). Anomalous vertebral arteries in the extra- and intraosseous regions of the craniovertebral junction visualized by 3-dimensional computed tomographic angiography: analysis of 100 consecutive surgical cases and review of the literature. Spine (Phila Pa 1976).

[9] Shimizu T, Koda M, Abe T (2021). Correlation between osteoarthritis of the atlantoaxial facet joint and a high-riding vertebral artery. BMC Musculoskelet Disord.

[10] Vaněk P, Bradáč O, de Lacy P, Konopková R, Lacman J, Beneš V (2017). Vertebral artery and osseous anomalies characteristic at the craniocervical junction diagnosed by CT and 3D CT angiography in normal Czech population: analysis of 511 consecutive patients. Neurosurg Rev.

[11] Kosnik-Infinger L, Glazier SS, Frankel BM (2014). Occipital condyle to cervical spine fixation in the pediatric population. J Neurosurg Pediatr.

[12] Ruetten S, Hahn P, Oezdemir S, Baraliakos X, Merk H, Godolias G, Komp M (2018). Full-endoscopic uniportal odontoidectomy and decompression of the anterior cervicomedullary junction using the retropharyngeal approach. Spine (Phila Pa 1976).

[13] Ruetten S, Hahn P, Oezdemir S, Baraliakos X, Merk H, Godolias G, Komp M (2018). The full-endoscopic uniportal technique for decompression of the anterior craniocervical junction using the retropharyngeal approach: an anatomical feasibility study in human cadavers and review of the literature. J Neurosurg Spine.

[14] Ohara Y, Uchikado H, Hara T, Nojiri H, Abe E, Kikuchi N, Kimura T (2023). Endoscopic approach for a difficult cervical area: fully endoscopic uniportal transcervical approach for ventral pathologies of the craniovertebral junction. J Minim Invasive Spine Surg Tech.

